# Comparative Analysis of Chemical Profile, Antioxidant, *In-vitro *and *In-vivo *Antidiabetic Activities of *Juniperus foetidissima *Willd. and *Juniperus sabina *L.

**Published:** 2017

**Authors:** Nilüfer Orhan, Didem Deliorman Orhan, Alper Gökbulut, Mustafa Aslan, Fatma Ergun

**Affiliations:** a *Department of Pharmacognosy, Faculty of Pharmacy, Gazi University, Ankara, Turkey.*; b *Department of Pharmacognosy, Faculty of Pharmacy, Ankara University, Ankara, Turkey.*

**Keywords:** Amentoflavone, Antioxidant, Antidiabetic, *Juniperus foetidissima*, *Juniperus sabina*

## Abstract

Fruit and leaves of junipers are commonly used internally as tea and pounded fruits are eaten to lower blood glucose levels in Anatolia. Thus, we aimed to evaluate antidiabetic and antioxidant potential and the chemical profile of *Juniperus foetidissima* Willd. and *J. sabina* L. in this study. *In-vitro* antidiabetic activities of leaf and fruit extracts were examined by their inhibitory activity on *α*-glucosidase and *α*-amylase enzymes. Then, *in-vivo* antidiabetic activities of leaf and fruit extracts of *Juniperus *species were investigated on streptozotocin-induced diabetic rats. Additionally, antioxidant activities (phosphomolybdenum, ferric-reducing antioxidant power and ABTS radical scavenging activity assays), phytochemical screening tests and high performance liquid chromatography analysis (HPLC) were done. *In-vitro* enzyme inhibitory effects of the extracts were supported by the results of* in-vivo *antidiabetic activity studies. Phytochemical screening tests indicated presence of flavonoids, tannins, terpenoids and carbohydrates in the extracts. Amentoflavone was identified as the major compound in the extracts and content of amentoflavone was determined. As a result, *Juniperus* extracts and its active constituents might be beneficial for diabetes and its complications.

## Introduction

*Diabetes mellitus* is a chronic disease that occurs either when the pancreas does not produce enough insulin or when the body cannot effectively use the insulin it produces. According to the report of WHO, an estimated 1.5 million deaths were directly caused by diabetes in 2012 and more than 80% of diabetes deaths occur in low- and middle-income countries (1). 

All around the world, fruit and leaves of several *Juniperus* species (Cupressaceae) are used as remedies against common cold, urinary infections, urticaria, dysentery, hemorrhage, rheumatic arthritis, to relieve menstrual pain, as diuretic, carminative, stomachic and antiseptic (2-5). On the other hand, infusion and decoctions of *Juniperus* fruits and leaves are used internally and pounded fruits are eaten for diabetes in Turkey as folk remedy (6, 7). 

The present study was performed to compare *in-vitro* (*α*-amylase and *α*-glucosidase enzyme inhibitory) and *in-vivo* antidiabetic effects of two *Juniperus *species and to determine their antioxidant potential and active components. Antioxidant activities of the extracts were determined by phosphomolybdenum, ferric-reducing antioxidant power and ABTS radical scavenging activity assays. Additionally, preliminary phytochemical tests were conducted on the extracts to define the secondary metabolites of the leaf and fruit extracts. Finally, high performance liquid chromatography (HPLC) analysis was done to determine the major compounds in the extract and to evaluate their amounts.

## Experimental

*Plant Materials*

Leaves and fruits of *Juniperus foetidissima* Willd. and *Juniperus sabina *L. were collected in October 2007 from Yozgat, Turkey. The plant was identified by N. Orhan and voucher specimens (GUEF 2619 and GUEF 2618) are stored in the Herbarium of Gazi University, Faculty of Pharmacy. 

*Extraction of Material*

The chopped dried fruits and leaves (100 g) were extracted with ethanol 80% (2 L) by mixer for 8 h individually. The day after, the extract was filtrated and the residue was extracted by the same procedure with ethanol again. The filtrates were pooled and evaporated to yield dry extracts under reduced pressure. After evaporation, yield of the extracts (w/w) were calculated as; *J. foetidissima *leaf ethanol extract: 32.5 %, fruit ethanol extract: 22.5 %, *J. sabina* leaf ethanol extract: 27.6 %, fruit ethanol extract: 19.8 %.

*In-Vitro Antidiabetic Activity Studies*

*Assay for α-Glucosidase Inhibitory Activity*

The method of Lam *et al*. (2008) was used to evaluate *α*-glucosidase inhibitory activity (8). *Bacillus stearothermophilus* originated *α*-Glucosidase type IV enzyme (Sigma Co., St. Louis, USA) was dissolved in phosphate buffer (0.5 M, pH 6.5). Extracts were dissolved in ethanol at different logarithmic concentrations (3000, 1000, and 570 µg/mL). The enzyme solution and test extracts were preincubated in a 96-well microtiter plate for 15 min at 37 °C. Then, 20 mM *p*-nitrophenyl-*α*-*d*-glucopyranoside (NPG), (Sigma) was added to the wells. The microtiter plate was incubated at 37 °C for 35 min. The increase in the absorption at 405 nm due to the hydrolysis of NPG by α-glucosidase was measured by an ELISA (VersaMax, Molecular Devices, USA) reader. Acarbose (Bayer Group, Turkey) was used as positive control. 

*Assay for α-Amylase Inhibitory Activity*

The method of Ali *et al*. (2006) was used to determine the *α*-amylase inhibitory activity of the selected *Juniperus *species (9). Porcine pancreatic *α*-amylase type VI (EC 3.2.1.1, Sigma) was dissolved in distilled water. Potato starch (0.5 %, w/v) in phosphate buffer (pH 6.9) was used as substrate solution. Plant extract was dissolved in DMSO at logarithmic concentrations. After the addition of the enzyme solution, mixtures were incubated at 37 °C for 3 min. Then, substrate solution was added and the mixtures were incubated at 37 °C for 5 min. DNS colour reagent solution (96 mM 3,5-dinitrosalicylic acid, 5.31 M sodium potassium tartrate in 2 M NaOH) was added to the mixtures and the tubes were put into a 85 °C heater. After 15 min, distilled water was added to the tubes and the tubes were cooled on ice. Absorbances of the mixtures were read at 540 nm. Acarbose was used as the positive control. Standard maltose calibration graph was prepared and the absorbance due to maltose generated was calculated according to the following formula: *A*_Control or Plant extract_=*A*_Test_−*A*_Blank.. _Inhibition percentages were calculated and given in Table 1.

**Table 1:** *α*-Glucosidase and α-amylase inhibitory activity of *Juniperus *ethanol extracts

**Plant Name**	**Plant Part**	**Extract**	***α*** **-Glucosidase Inhibitory Activity** **(Inhibition % ** **± S.D.)**			***α-*** **Amylase Inhibitory Activity ** **(Inhibition % ± S.D.)**		
			**3000 µg/mL**	**1000 µg/mL**	**570 µg/mL**	**3000 µg/mL**	**1000 µg/mL**	**570 µg/mL**
***Jf***	Fruit	EtOH	98.3 ± 1.9	89.6 ± 0.7	72.7 ± 1.2	41.1 ± 5.4	40.0 ± 2.1	3.5 ± 0.2
	Leaf	EtOH	>100	97.3 ± 0.3	88.6 ± 0.4	>100	65.1 ± 2.6	13.7 ± 2.6
***Js***	Fruit	EtOH	72.8 ± 1.1	48.3 ± 1.3	29.6 ± 0.8	39.6 ± 4.0	32.9 ± 1.4	29.6 ± 0.8
	Leaf	EtOH	98.1 ± 0.5	93.9 ± 1.9	87.6 ± 0.9	39.6 ± 7.0	26.4 ± 1.6	6.4 ± 0.8
**Acarbose**	**Concentration**		**100 µg/mL**	**30 µg/mL**	**10 µg/mL**	**100 µg/mL**	**30 µg/mL**	**10 µg/mL**
	**Inh. % ** **± S.D.**		97.1 ± 0.2	95.0 ± 0.3	90.9 ± 0.4	76.0 ± 3.5	28.0 ± 9.8	-

*Jf: Juniperus foetidissima, Js: J. sabina*

*In-Vivo Antidiabetic Activity Studies*

*Preparation of test samples*

The extracts were suspended in 0.5% aqueous carboxymethylcellulose (CMC-suspension in distilled water) prior to oral administration to animals. Glipizide [10 mg/kg, body weight (b.w.)] was used as the reference drug. Glipizide was purchased from Sigma (G117-1 g, St. Louis, MO 63103 USA). Animals in the control group received only the vehicle 0.5% aqueous carboxymethylcellulose (10 mL/kg, b.w.). 

*Animals*

Male Wistar-albino rats (150-200 g) purchased from the Animal House of Gazi University (Ankara, Turkey) were used in the experiments. Prior to the experiments, rats were fed with standard food for one week in order to adapt to the laboratory conditions. Institutional Animal Ethical Committee of the Gazi University approved (G.Ü.ET-06.087) the experimental protocol used in the present study.

*Determination of the blood glucose levels*

The rats were fasted 12 h before the determination of blood glucose levels, but allowed free access to water. Blood glucose concentrations (mg/dL) were determined using an Ascensia-Elite commercial test (Serial No. 9123232, Bayer), based on the glucose oxidase method. Blood samples were collected from the tip of tail at the defined time patterns.

*Effect in diabetic animals (non-insulin dependent diabetes model)*

Experimental diabetes was induced by intraperitoneal (i.p.) injection of streptozotocin (STZ) at a dose of 65 mg/kg b.w. dissolved in distilled water (1 mL/kg). Three days after the injection, the blood glucose levels were measured and the animals with blood glucose levels higher than 300 mg/dL were considered as diabetic. 

For determination of antidiabetic activity, diabetic animals were fasted for 6 h (water ad libitum). Test samples were given orally using oral gastric gavages. The blood glucose concentrations were measured in all animals at the beginning of the study and the measurements were repeated 1/2, 1, 2, 4 h and 6 h after the initial of the experiment.

*In-Vitro Antioxidant Activity Studies*

*Total antioxidant activity by phosphomolybdenum assay*

This assay is based on the reduction of Mo (VI) to Mo (V) by the sample and the subsequent formation of a green phosphate/Mo (V) complex at acidic pH. *Juniperus *ethanol extracts were added to test tubes containing distilled water and moybdate reagent solution. Vortexed tubes were incubated at 90 ºC for 90 min. Then, the tubes were cooled to room temperature and the absorbances of the samples were measured at 695 nm. Results were expressed as ascorbic acid equivalent (AAE) (10).

*Ferric-reducing antioxidant power*

The reducing power of the extracts was determined by the reducing power assay of Oyaizu (1986) with slight modifications (11). Different logarithmic concentrations of the extracts (3,1, and 0.57 mg/mL) and ascorbic acid as reference were mixed with phosphate buffer (0.2 mol/L, pH 6.6) and K_3_Fe(CN)_6_. Tubes were incubated at 50 ºC for 20 min, then trichloroacetic acid was added and the mixture was vortexed. The following centrifugation, the supernatant was mixed with distilled water and FeCl_3_ and the absorbance at 700 nm was measured. The analyses were run in three replicates and the results were averaged.

*Assay for scavenging activity of ABTS radical cation*

ABTS radical cation (ABTS·+) scavenging assay was achieved by using the spectrophotometric methods of Re *et al*. (1999) and Meot-Duros *et al*. (2008) with slight modifications (12, 13). ABTS (7 mM) was dissolved in distilled water and the ABTS radical cation was generated by adding 2.45 mM potassium per-sulfate. The radical production was completed after incubation for 16 h in the dark at 20°C. Absorbance of ABTS solution was adjusted to 0.7 ± 0.02 at 734 nm by the addition of phosphate buffer solution (PBS) at pH 7.4. 1 mL diluted ABTS solution was added to 10 μL of extract (PBS or Trolox). Samples were vortexed and their absorbances were read versus PBS blank at 734 nm. Trolox was used as the positive control. 

*Phytochemical screening*

Preliminary phytochemical composition of ethanol extracts of *J. foetidissima* and *J. sabina* fruits and leaves was analyzed for their chemical constituents. Phytochemical screening was done as described in literature (14, 15). Following reagents and chemicals were used: Alkaloids with Dragendroff’s reagents, flavonoids with metalic magnesium plus HCl, phenolics with Ferric chloride reagent, cardiac glycosides with Liberman’s test and Keller Killiani test, anthraquinones with Borntrager’s reaction, saponins with the ability to produce suds, reducing sugars with Fehling’s reagent, triterpene steroids with sulphuric acid reagent. Terpenoids were visualized by anisaldehyde-sulphuric acid on TLC plates.

*Qualitative and quantitative analyses of phenolic compounds using RP-HPLC-DAD*

The qualitative and quantitative analyses of the phenolic compounds in the fruits and leaves of the species were performed according to the following procedure: Chlorogenic acid (C3878), caffeic acid (C0625), ferulic acid (128708), *p*-coumaric acid (C9008), myricetin (70050), quercetin (Q0125), luteolin (L9283), apigenin (10798), amentoflavone (40584) and umbelliferone (H24003) were purchased from Sigma-Aldrich, Germany. Protocatechuic acid was purchased from HWI Analytik GmbH, Germany. All other chemicals were analytical grade and obtained from either Sigma or Merck. The analysis was performed with a LC system consisting of a HP Agilent 1260 series quaternary pump, degasser and photo-diode array detector. The samples were injected using HP Agilent 1260 Autosampler with a thermostatted column compartment on a ACE column (5 μm, 250 mm X 4.6 mm) at 30°C. The system was controlled and data analysis was performed with Agilent ChemStation software. All the calculations concerning the quantitative analysis were performed with external standardization by measurement of the peak areas. Gradient elution was applied with a flow rate of 0.8 mL/min and column temperature was set to 30 °C. 

The mobile phase was a mixture of trifluoroacetic acid 0.1% in water (solution A), trifluoroacetic acid 0.1% in methanol (solution B), and trifluoroacetic acid 0.1% in acetonitrile (solution C). The composition of the gradient was (A:B:C), 80:12:8 at 0 min, 75:15:10 at 8 min, 70:18:12 at 16 min, 65:20:15 at 24 min, 50:35:15 at 32 min, 25:60:15 at 40 min and 80:12:8 at 45 min. 

All solvents were filtered through a 0.45 μm filter before use and degassed in an ultrasonic bath. From each solution and sample 10 μL was injected into the column and the chromatograms were recorded from 200 to 400 nm. Standard solutions were analyzed and three-dimensional chromatograms (wavelength; time; absorbance) were obtained to select the optimum wavelength for detection of the phenolics with maximum sensitivity. Quantification was performed by measuring at 330 nm for amentoflavone and umbelliferone using a photo-diode array detector. The chromatographic run time was 45 min. The duration between runs was 2 min.

*Calibration*

Six different concentrations of amentoflavone and umbelliferone were prepared in methanol ranging between 1-1000 μg/mL, 0.2-1000 μg/mL, respectively. Triplicate 10 μL injections were made for each standard solution to see the reproducibility of the detector response at each concentration level. The peak areas obtained from injections were plotted against the concentrations to establish the calibration graphs. The quantification of amentoflavone and umbelliferone was performed in reference to the obtained calibration curves.

*Limits of detection and quantification*

Limits of detection (LOD) were established at a signal to noise ratio (S/N) of 3. Limits of quantification (LOQ) were established at a signal to noise ratio (S/N) of 10. LOD and LOQ were experimentally verified by the nine injections of reference compounds in LOQ concentrations.

*Precision*

The precision of the method (within–day variations of replicate determinations) was checked by injecting nine times of amentoflavone and umbelliferone at the LOQ levels. The area values were recorded and RSD% was calculated.

*Recovery*

The spike recovery was carried out by the standard addition method. For the determination of the recovery, three different concentrations of amentoflavone and umbelliferone (0.01, 0.1, and 1 mg/mL) were added to the extracts. In each additional level, six determinations were carried out and the mean value of recovery percentage was calculated.

*Statistical Analysis*

Values were presented as means ± standard error of the mean (S.E.M.). Statistical differences between the treatments and the controls were tested by one-way analysis of variance (ANOVA) followed by the Student-Newman-Keuls test using the MS-DOS software (GraphPad InStat statistical program). Linear regression analyses were done by using Microsoft Excel. A difference in the mean values of p<0.05 was considered to be statistically significant. All *in-vitro* experiments were carried out with minimum three replicates.

## Results

Within the context of present study, antidiabetic activities of ethanol extracts from the fruits and leaves of *J. foetidissima *and* J. sabina *were investigated by using both *in-vitro* and *in vivo* techniques in order to evaluate their folk medicine practices in Turkey. *α*-Glucosidase inhibitory activities of *Juniperus *leaf and fruit extracts were tested at 3 different logarithmic concentrations and their activities were compared with the reference drug Acarbose (Table 1). It was surprising to find that all extracts have promising *α*- glucosidase inhibitory activity. Leaf extracts were more active than the fruit extracts and *J. foetidissima* leaf ethanol extract was found to have the highest activity (>100-88.6 %) among all tested extracts. 

On the other hand, *α*-amylase inhibitory activities of *Juniperus *extracts were tested and the results were given in Table 1. All the tested extracts were found to have moderate enzyme inhibitory activity. *J. foetidissima* leaf ethanol extract was the most active extract again as in *α*-glucosidase inhibition assay. Its inhibitory activity at 1000 µg/mL (65.1%) was close to inhibitory activity of reference drug Acarbose (76.0%).

Based on the striking results obtained from the *in-vitro* enzyme inhibitory activity tests, *in-vivo* antidiabetic activities of the extracts were evaluated on diabetic rats. The extracts were administrated to STZ-induced diabetic rats at 500 and 1000 mg/kg dose to determine their acute effects on blood glucose concentrations. Changes in the blood glucose levels of each group were followed during a 6 h period. As demonstrated in Table 2, reference drug Glipizide (10 mg/kg) did not possess a potent activity (1.9-11.5%) on STZ induced diabetic rats. However, the leaf extracts showed a slight and continuous activity between 0.2-13.3% compared to diabetic control group. Although the antidiabetic activity of the *Juniperus *extracts may be considered to be mild, it is remarkable and deserves attention. Because the initial blood glucose levels of diabetic animals was so high (377.8-381.9) showing that the animals was in severe form of diatebes. The blood glucose lowering effect profile of leaf extracts is similar to profile of the reference drug, thus a promising reduction is observed 1-2 h after the beginning of the experiment. The results of our *in-vitro* studies supported findings of our in *in-vivo *studies and the antidiabetic activity of the *J. foetidissima* leaf extract was found to be the highest. There was a surprising result that *J. sabina* leaf extract caused death (33.3%) in both doses (500 and 1000 mg/kg). 

**Table 2:** Antidiabetic effects of *Juniperus* leaf ethanol extracts in STZ-diabetic rats

**Group**	**Dose** **(mg/kg)**	**Blood Glucose Concentration (mg/dL) Standart Error of the Mean (Inhibition %)**					
		**Initial**	**½ h**	**1 h**	**2 h**	**4 h**	**6 h**
**Control**	-	381.528.0	388.4 ± 9.9	389.4 ± 5.1	356.4 ± 5.5	311.1 ± 10.7	296.0 ± 9.1
**Glipizide**	10	381.5±29.8	380.9 ± 4.9 (1.9 %)	355.6 ± 5.7** (8.7 %)	315.5 ± 4.5*** (11.5 %)	284.1 ± 13.1* (8.7 %)	283.4 ± 21.1 (4.3 %)
***Jf***	500	381.9±26.7	357.3 ± 31.7* (8.0 %)	361.1 ± 24.8 (7.3 %)	365.1 ± 11.2	311.6 ± 13.3	294.3 ± 15.1 (0.6 %)
	1000	377.8±23.9	363.9 ± 17.4 (6.3 %)	337.8 ± 6.1*** (13.3 %)	333.5 ± 15.3* (6.4 %)	310.4 ± 14.2 (0.2 %)	285.0 ± 9.8 (3.7 %)
***Js***	500	380.8±26.0	361.7 ± 9.6 (6.9 %)	345.0 ± 7.2*** (11.4 %)	352.8 ± 16.5 (1.0 %)	300.6 ± 16.5 (3.4 %)	314.6 ± 7.5
	1000	381.0±8.3	362.4 ± 21.5 (6.7 %)	349.7 ± 4.5*** (10.2 %)	339.3 ± 29.3 (4.8 %)	299.5 ± 24.0 (3.8 %)	300.5 ± 16.6

*Jf: Juniperus foetidissima, Js: J. sabina, *

* : p<0.05,

** : p<0.01,

*** : p<0.001

Results of *Juniperus* fruit extracts on diabetic rats after oral administration is given in Table 3. Only *J. foetidissima* fruit ethanol extract at 500 mg/kg dose significantly lowered blood glucose levels of diabetic animals. Its effect was similar to reference drug Glipizide.

**Table 3:** Antidiabetic effect of *Juniperus* fruit ethanol extracts in STZ-diabetic rats

**Group**	**Dose** **(mg/kg)**	**Blood Glucose Concentration (mg/dL) Standart Error of the Mean (Inhibition %)**					
		**Initial**	**½ h**	**1 h**	**2 h**	**4 h**	**6 h**
**Control**	-	372.8 ± 24.0	376.3 ± 5.4	382.2 ± 4.2	350.7 ± 7.1	311.3 ± 6.1	313.2 ± 3.9
**Glipizide**	10	372.0 ± 29.5	338.4 ± 4.0*** (10.1 %)	342.1 ± 4.9*** (10.5 %)	323.1 ± 10.7** (7.9 %)	307.4 ± 10.8 (1.3 %)	309.4 ± 10.7 (1.2 %)
***Jf***	500	376.6 ± 39.7	371.5± 19.9 (1.3 %)	341.3 ± 7.6*** (10.7 %)	325.5 ± 22.7* (7.2 %)	277.6 ± 4.1 (10.8 %)	306.0 ± 19.6 (2.3 %)
	1000	380.8 ± 28.9	393.0 ± 17.2	393.8 ± 12.2	365.4 ± 14.6	306.0 ± 21.8 (1.7 %)	287.3 ± 12.6 (8.3 %)
***Js***	500	382.8 ± 35.5	386.6 ± 20.6	413.2 ± 26.7	339.0 ± 20.97 (3.4 %)	311.2 ± 35.9	335.6 ± 46.5
	1000	372.1 ± 22.2	397.0 ± 25.3	406.8 ± 24.5	348.1 ± 17.4 (0.7 %)	293.7 ± 24.3 (5.7 %)	283.7 ± 21.2 (9.4 %)

*Jf: Juniperus foetidissima, Js: J. sabina, *

* : p<0.05,

** : p<0.01,

*** : p<0.001

Antioxidant potential of ethanol extracts was also evaluated by *in-vitro* antioxidant activity tests. Phosphomolibdenum assay was used to determine total antioxidant activity of the extracts and the results are given as ascorbic acid equivalent/g extract (Table 4). Antioxidant capacities of leaf extracts were higher than the fruit extracts’. *J. sabina* leaf extract was the most active one and its activity (556.2 AAE) was determined much more than all tested reference antioxidants (BHA, BHT, Gallic acid and Trolox) at 3000 µg/mL concentrations. According to the results of ferric reducing power assay, all the extracts exhibited high absorbance values at 3000 µg/mL concentrations, close to ascorbic acid which was used as a reference. Ferric reducing power of leaf extracts was higher than that of fruit extracts and *J. sabina* leaf ethanol extract was the most active extract. The highest ABTS radical scavenging activity was observed by the *J. foetidissima* leaf extract whereas other extracts showed similar radical scavenging activities ranging from 50.0 to 1.0%. 

**Table 4:** Antioxidant activities of *Juniperus *ethanol extracts

**Plant Name/ Reference**	**Plant Part**	**Tot. Ant. Act.** **(AAE **± **S.D.)**	**Reducing Power** **(Absorbance **± **S.E.M**.**)**			**ABTS Radical Scavenging Activity** **(Inhibition % ± S.D.)**		
		**3000** ** µg/mL**	**3000** ** µg/mL**	**1000** ** µg/mL**	**570** ** µg/mL**	**3000** ** µg/mL**	**1000** ** µg/mL**	**570** ** µg/mL**
***Jf***	Fruit	169.5 ± 28.8	0.1641 ± 0.0015	0.1384 ± 0.0031	0.1063 ± 0.0017	52.0 ± 1.7	13.8 ± 1.0	5.8 ± 1.6
	Leaf	287.5 ± 6.6	0.1684 ± 0.0072	0.1460 ± 0.0007	0.1237 ± 0.0033	89.0 ± 0.2	62.6 ± 0.2	10.3 ± 1.0
***Js***	Fruit	149.8 ± 11.8	0.1427 ± 0.0014	0.0720 ± 0.0023	0.0430 ± 0.0018	25.3 ± 1.7	6.3 ± 1.7	1.0 ± 0.3
	Leaf	556.2 ± 46.6	0.1600 ± 0.0079	0.0920 ± 0.0013	0.0560 ± 0.0016	39.6 ± 0.9	10.9 ± 1.1	6.6 ± 1.8
**Trolox**	-	382.5 ± 17.0	NT	NT	NT	NT	NT	NT
**Gallic Acid**	-	38.4 ± 5.6	NT	NT	NT	>100	99.9 ± 0.1	99.3 ± 0.2
**BHT**	-	54.7 ± 8.7	NT	NT	NT	NT	NT	NT
**BHA**	-	159.6 ± 3.3	NT	NT	NT	NT	NT	NT
**Ascorbic A.**	-	-	0.1868 ± 0.0016	0.1845 ± 0.0075	0.1812 ± 0.0084	NT	NT	NT

*Jf: Juniperus foetidissima, Js: J. sabina, *Tot. Ant. Act.: Total Antioxidant Activity, NT: Not tested

**Figure 1:**
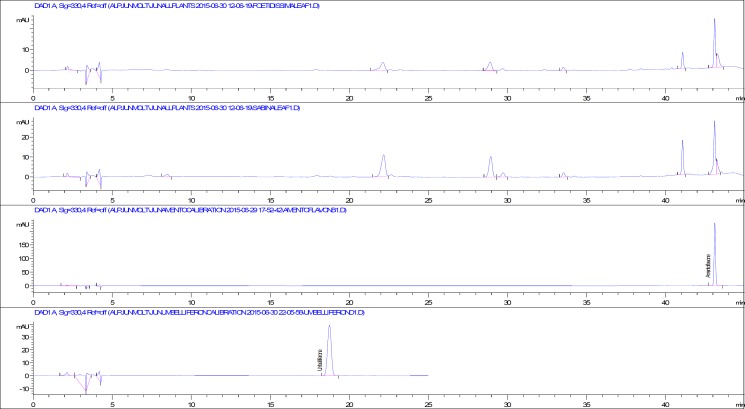
HPLC chromatograms of *J. foetidissima* and *J. sabina *leaf extracts, amentoflavone and umbelliferone

According to the results of phytochemical screening tests, flavonoids, phenolics, reducing sugars and terpenoids were found both in fruit and leaf extracts of selected *Juniperus *species. After that, a validated RP-HPLC method was used for the qualitative and quantitative analysis of phenolics in the *Juniperus* extracts. In previous studies, protocatechuic acid, chlorogenic acid, apigenin, quercetin glycosides, biflavonoids, amentoflavone and umbelliferone were reported from different *Juniperus* taxa grown in Turkey (16-18). Our results revealed that protocatechuic acid, chlorogenic acid, caffeic acid, ferulic acid, *p*-coumaric acid, myricetin, quercetin, luteolin and apigenin were not detected in any of the investigated *Juniperus* extracts. Among all the screened phenolic compounds only amentoflavone and umbelliferone were detected and quantified. HPLC chromatograms of the leaf extracts, amentoflavone and umbelliferone were given in Figure 1.

**Figure 2:**
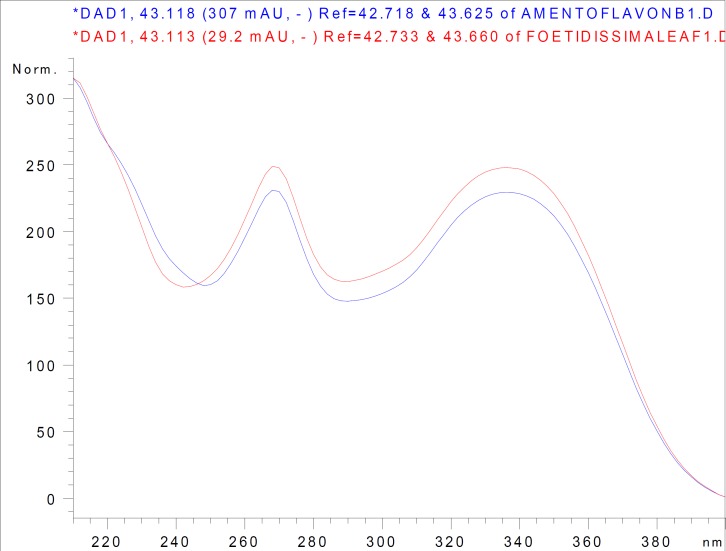
Overlaid UV spectra of amentoflavone in *J. foetidissima* leaf extract and authentic amentoflavone

In approximately 45 min, amentoflavone and umbelliferone were quantified after evaluating the retention times and UV spectra of the authentic phenolic compounds and the phenolic compounds in the extracts. For instance, the UV spectra of authentic amentoflavone and amentoflavone in the leaf extract of *J. foetidissima* were overlaid and both spectra fit wonderfully (Figure 2). 

The method was validated and fine results were obtained. The precision of the method was checked by injecting nine times of amentoflavone and umbelliferone at the LOQ levels and expressed as RSD%: 1.3558, 1.0326, respectively. Retention times, linear relationships between peak areas and concentrations, test ranges, LOD and LOQ values were given in Table 5. The recovery values for amentoflavone and umbelliferone fell within the ranges from 91.10 to 97.65% and 95.89 to 103.49%, respectively. The contents of amentoflavone and umbelliferone in the leaves and fruits of *Juniperus* species were given in Table 6. Amentoflavone, the major compound of the *J. foetidissima* and *J. sabina* leaves, was determined as 0.217±0.001 g/100g dw and 0.208±0.009 g/100g dw, respectively. *J. foetidissima* and *J. sabina* fruits contain amentoflavone as 0.044±0.002 g/100g dw and 0.027±0.003 g/100g dw, respectively. On the other hand, umbelliferone was determined only in the leaves while fruits were lacked of this coumarin compound. 

**Table 5:** Retention times, linear relationships between peak areas and concentrations, test ranges, LOD and LOQ

**Compound**	**Retention time** **(min)**	**Standard curve**	**R** ^2^	**Test range** **(µg/mL)**	**LOD (µg/mL)**	**LOQ (µg/mL)**
**Amentoflavone**	43.1	y=26854x-12.591	0.9999	1-1000	0.037	0.123
**Umbelliferone**	18.7	y=72936x-19.485	0.9993	0.2-1000	0.031	0.102

R.t.: Retention time, y: peak area; x: concentration (mg/mL); LOD= limit of detection S/N:3 (n=9); LOQ= limit of quantification S/N:10 (n=9).

**Table 6:** Amentoflavone and umbelliferone contents (g/100g dw) of fruits and leaves of *Juniperus *species

**Compound **	***Jf *** **fruit**	***Jf *** **leaf**	***Js *** **fruit**	***Js *** **leaf**
**Amentoflavone**	0.044 ± 0.002	**0.217 ± 0.001**	0.027 ± 0.003	**0.208 ± 0.009**
**Umbelliferone**	nd	0.011 ± 0.001	nd	0.013 ± 0.001

*Jf: Juniperus foetidissima, Js: J. sabina,* dw: dry weight, Results are expressed as mean ± SD (n=3); nd=not detected.

## Discussion

This researchis the first study on the *in-vivo *and* in-vitro *antidiabetic activities of *J. foetidissima* and *J. sabina *leaf and fruit extracts. Additionally, phenolic profile of these plants is presented for the first time. The results of our study revealed that extracts of both *Juniperus* species exhibit promising antidiabetic and antioxidant activities. Although *J. sabina* leaf extract has strong enzyme inhibitory activity it has toxic effects on living organisms as mentioned before (19). In order to determine the compounds responsible for the significant antidiabetic and antioxidant potential of *J. foetidissima* and *J. sabina *fruit and leaf extracts, we investigated the phenolic compound profile of the extracts using HPLC-DAD system. Most of the checked phenolics were not determined in the ethanol extracts except amentoflavone and umbelliferone. Amentoflavone was found as the major component of the both leaf extracts. 

*Juniperus* is an important genus widely distributed in the North hemisphere and it is mainly a source of sesquiterpenes, diterpenes, lignans, and flavonoids some of them with very promising biological activities as mentioned by Seca and Silva (4). Antidiabetic activities of different *Juniperus* species have been the subject of many studies as well as many other biological activities. Swanston-Flatt *et al*. (1999) and Sanchez de Medina *et al*. (1994) have worked on *J. communis, *and Aboul-ela *et al*. (2005) have worked on *J. phoenicia *extracts (20-22). Loizzo *et al*. (2007) evaluated *in-vitro *hypoglycaemic activity of *J.oxycedrus *ssp. *oxycedrus *berry and wood essential oils by the inhibition of *α*-amylase, wood oil was found to be active (23). Our group has investigated *in-vivo* antidiabetic activities of *J. oxycedrus* ssp. *oxycedrus* leaf and fruit extracts in detail and identified their active components (24, 25). Additionally, we have evaluated *in-vitro* antidiabetic activities of *J. communis *and *J. oxycedrus* leaf and fruit extracts and found that they have a spectacular enzyme inhibitory activity (26).

On the other hand, Asgary *et al*. (2013) investigated antioxidant and anti-glycation properties of essential oils from branchlets and fruits of *J. sabina. *Antioxidant effects were evaluated using low density lipoprotein oxidation, linoleic acid peroxidation and red blood cell hemolysis assays (27). Essential oils possessed antioxidant properties and promising effects were observed from the tested oils against hemoglobin (39.7-79.6%) and insulin (56.9-89.2%) glycation. On the other hand, the same research group (28) has evaluated antioxidant and anti-glycation properties of *J. foetidissima *branches and fruit essential oils. Essential oils have inhibited hemoglobin and insulin glycation significantly as well as their promising antioxidant activity. 

Na *et al*. (2007) has investigated the protein tyrosine phosphatase 1B (PTP1B) inhibitory activity of amentoflavone and it has inhibited PTP1B enzyme with an IC50 value of 7.3 µM (29). Additionally, treatment of 32D cells over expressing the insulin receptor with amentoflavone resulted in a dose-dependent increase in tyrosine phosphorylation of the receptors. Results have proved that amentoflavone may enhance insulin-induced intracellular signaling possibly through inhibition of PTP1B activity. On the other hand, Kim *et al*. (2000) has presented that amentoflavone has strong inhibitory activity against yeast *α*-glucosidase and porcine pancreatic *α*-amylase (30). 

Thus, it is clear that amentoflavone has significant inhibitory activity on carbohydrate digestive enzymes as well as its positive effects on insulin resistance. For this reason, the antidiabetic activity of the extracts should be attributed to amentoflavone which was determined in serious amounts in all the extracts, especially in the leaf extracts. The more *α*-glucosidase inhibitory activity of the leaf extracts when compared to fruit extracts could be explained by the higher amount of amentoflavone in the leaf extracts. 

## Conclusion

In conclusion, the results of *in-vivo* and *in-vitro* studies proved that *J. foetidissima* leaves have a promising antidiabetic activity compatible with its folkloric usage. Further studies may be planned to see the long term effects of the *J. foetidissima* extracts on diabetes mellitus.
